# CANCER IN OTHER WORDS? THE ROLE OF METAPHOR IN EMOTION DISCLOSURE IN CANCER PATIENTS

**DOI:** 10.1111/bjp.12023

**Published:** 2013-04-17

**Authors:** Anne Lanceley, Jill Macleod Clark

**Affiliations:** Dept. of Women's Cancer, the UCL Elizabeth Garrett Anderson Institute for Women's Health, University College London, Medical School Building74 Huntley StreetLondon WC1E 6AU; FRCN is Deputy Dean of the Faculty of Medicine, Health and Life Sciences, University of Southampton, South Academic Block, Southampton General HospitalTremona Road, Southampton

**Keywords:** nurse–patient communication, cancer, emotion, metaphor, literary criticism

## Abstract

Despite evidence that nurses may play a crucial part in the wellbeing and recovery of cancer patients by facilitating their expression of feelings, research is lacking into the emotional content of nurse–patient talk and patients' use of language in emotion disclosure. In this study, 23 participating nurses in a variety of cancer care settings were asked to tape-record their conversations with patients during daily care. A data set of 60 nurse–patient conversations was collected. Individual expression of emotion by patients was identified through interpretive literary analysis within a framework of psychodynamic theory. Overall the picture of emotion disclosure was intense. In particular, patients' use of metaphor and figurative language to express their distress was powerful and pervasive. Participating nurses demonstrated responsive skills but their responses to figurative expression were often problematic.

The study provides evidence of unconscious processes in nurses' work and advocates career-long psychoanalytically informed supervision for nurses to better support them in challenging dialogue with cancer patients. Research is needed to evaluate the impact of supervision on communications with cancer patients to ensure patients have access to appropriate emotional supportive and care.

## Introduction

Cancer can change nearly all aspects of a person's life. Analysing how patients talk about, and make sense of, their own and others' experience of cancer illness has gained currency over the last 30 years, reflecting increased recognition of the affective relationship between health-care professional and cancer patient as the foundation of sensitive, appropriate care and communication (Kennifer *et al*., 2009; National Cancer Action Team, 2010; National Institute for Clinical Excellence, 2004). Patient complaints about poor communication (Department of Health, 2002) and debate about the role of psychological therapies in cancer care have also prompted research. Indeed a number of studies have examined whether psychological support improves survival rates. For example, Watson *et al*. (2005) evaluated an approach which encouraged emotional expression and a ‘fighting spirit’. Although there was no survival benefit the psychological therapy enhanced quality of life. Evidence that emotional and psychological support provides a sense of wellbeing and helps individuals cope with the trauma of cancer is a rationale for this study (Goodwin *et al*., 2001; LeMay & Wilson, 2008).

To date only a handful of studies have offered in-depth analysis of cancer patient disclosure, and they have largely focused on enumerating professionals' communication skills in identifying patients' worries and fears rather than on patients' contribution to the conversation (Maguire *et al*., 1996; Jansen *et al*., 2010). The research shows that nurses, consciously or not, tend not to accept patients' feelings, and lack skill in managing them in conversation (Schofield, Green & Creed, 2008; Sheldon *et al*., 2009; Oguchi *et al*., 2011). These research findings have spurred efforts to improve nurses' communication practices through cognitive skills-based training (Uitterhoeve *et al*., 2009). While intervention studies have demonstrated improvement after training and communication workshops, there is little evidence that it is sustained in practice (Parle, Maguire & Heaven, 1997). Our research throws light on the reasons. We identify the opportunities patients take in conversation to express their feelings, how they articulate them to the nurses, and the nature of their disclosures, elucidating the links between personal meaning and emotional expression. In so doing we consider both the experience of the patients and that of the nurse caring for them.

## Theoretical Framework

The correspondence between the emotional experience of illness and its expression through talk has occupied psychoanalytic thinkers as well as literary theorists and linguistic philosophers. From the psychoanalytic view point, the onset of cancer illness is a ‘psychic trauma’ evoking previously hidden fears and conflicts and causing anxiety (Erskine & Judd, 1994, p. 87). Rational thinking becomes distorted, giving rise to apprehension about fundamental threats to bodily integrity; isolation and loss due to hospitalization and being unwell; and the possibility of death (Emanuel, 2000). The use of words and images is particularly significant, pointing to the way in which a person consciously constructs the cancer threat, and unconsciously stirs hidden thoughts and feelings (Goldie, 2005). In our approach unconscious feelings are regarded as lying not outside the realm of language but contained within it; words are considered major symbolic forms (Lacan, 1977). The term symbolic here implies a form of imaginative self-extension from the domain of private experience: metaphorical and symbolic language is the vehicle for the creative expression of personal meaning and associated feelings. Language is used both to pin things down and, symbolically, to create a further dimension. Lakoff and Johnson's seminal work, *Metaphors We Live By* (1980), provides evidence that metaphor is responsible for sense-making and expression of feeling.

Since the growth of the capacity for symbol formation is crucial for making sense of emotional experience we also refer to the work of Klein and Segal. Klein, following Freud and Jones, showed through her work with children the development process of a symbolically functioning mind, the symbolic significance of play, and how sublimation relies on the capacity to symbolize (Klein, 1930). The endeavour to replace lost experience is a major spur to symbol formation in a process which assumes both the ability to represent an absent object and an ability to recognize that the symbol is not the symbolized object. In her seminal paper, ‘Notes on symbol formation’, Segal (1957) distinguished between more primitive and developed forms of symbolic function: she considered that only when separation and separateness are accepted does the symbol become the representative of the object rather than being equated with the thing symbolized.

Our interpretive analysis is also informed by a post-structuralist perspective which sees language as not transparently reflecting reality but as constructing and organizing it (Derrida, 1982). Meaning is taken to be inherently unstable, in part due to the figurative nature of language in which metaphor ‘deploys … initial experience into an infinity of associated experiences that spring from it’ (de Man, 1987, p. 104). Within this post-structuralist frame, interpretations offered are not the only ones possible and require subsequent exploration with the patient.

## Study Design

Ethical approval to conduct the study at two London cancer centres was obtained from the relevant National Health Service Research Ethics Committees. Sixty conversations between 21 nurses and 46 female and 14 male patients aged 20–81 with different cancers were recorded on audio-tape. Some patients were receiving aggressive curative therapies; others were having palliative treatment for symptom control or were being nursed on the wards while dying. Twenty-one cancer nurses, with varying degrees of clinical experience and communication skills training agreed to participate. We asked each participating nurse to tape-record two to three conversations with patients. The choice of which conversation to record was left to the nurse who would first obtain the patient's verbal consent. A pocket-size ‘Walkman'-style recorder with an internal microphone produced a clear, easily transcribed recording. Once the conversation had been recorded and its content known to both nurse and patient, the nurses made sure the patients did not wish to revoke consent before asking them to provide written consent to use the material. Nurses who gave written consent to participate were adequately supported during the study, for we recognized that they were required to share with us, and make ‘public’ in verbatim transcripts dimensions of their practice that were ordinarily private.

## Analysis

Data were first subjected to traditional discourse analysis, summarizing how sequences of emotion disclosure were brought about, managed and responded to by patient and nurse (Jaworski & Coupland, 2006). This analysis will be reported in a forthcoming paper. Although it revealed the pervasiveness of patients' emotional expression and how it was structurally managed, it did not bring out individual, personal ways in which cancer patients expressed their feelings, nor did it adequately account for the difficulties nurses faced in their conversations. Our perception of this first stage was that further analysis using a different method was needed to understand the interaction more fully.

Our second analysis applied techniques of literary criticism within a psychodynamic framework informed by theories of metaphor and emotion expression described above. Criticism of literary texts emphasizes individual sensibility and aims ‘to attain a peculiar completeness of response…’ (Leavis, 1952, p. 213). This involves sustained attention to the way the text achieves its effect and the kind of achievement involved. We analysed transcripts step-by-step to elucidate possible meanings for personal expression and language use following Richards's *Practical Criticism* (1929). This meant:

1. close reading to give full weight to each word;
2. assessment of tone and sensibility in a particular passage and ‘placing’ it before moving to the next;
3. detecting ambiguities;
4. characterizing the kind and quality of the imagery;
5. determining the precise degree of evocation of particular figures and metaphors;
6. meeting of co-authors to discuss variation in interpreting data.

Bringing out the tensions and contradictions and different layers of meaning within the transcripts (Parker, 1992), we sought to decide whether some readings make more sense than others once we had a context for them. Insights gained from nurse-participants during one-to-one interviews allowed us to incorporate important clinical impressions into our analysis (Mays & Pope, 2000).

## Results

A key finding was a powerful personal imagery used by patients to express feelings to nurses. Three data sequences from 54 hours of recorded nurse–patient conversations are analysed here to demonstrate the emotional meaning expressed in language of three different patients' individual experience of cancer illness and treatment. Pseudonyms are used for patients and nurses. Speech delivery is indicated as follows:

1. Short pauses are marked (.).
2. Seconds of silence are given in brackets, e.g. (3).
3. A full stop before a word or sound indicates an audible intake of breath, e.g. .no.
4. One or more colons indicate drawing-out of a preceding vowel sound, e.g. fi::ne.
5. Non-verbal sounds are described in brackets, e.g. (cries), (laughs).
6. Words emphasized by participants are underlined, e.g. talk

## Patient One

### Background

Doris, aged 75, is married and has five children. Throughout her married life she has lived in the same flat, with mainly the same neighbours, in the same inner-city borough. Doris's husband has chronic leg ulcers and has been unemployed for some years. He has a history of alcohol abuse, and the marital relationship is antagonistic. Doris's unemployed youngest son lives with them; her youngest daughter lives in the flat above. Doris had attended a specialist hospital for a rare skin condition for two years before her lung cancer was suspected after a routine chest X-ray, a month before the recorded conversation. She was immediately referred to a cancer centre where a bronchoscopy with biopsy revealed an adenocarcinoma of her lung with mediastinal secondaries. A fortnight before the conversation Doris experienced acute pain at home and was admitted to hospital as an emergency. Opiate analgesia alleviated her pain and a five-day course of palliative radiotherapy further eased the pain and swallowing difficulties she had been experiencing. The Senior House Officer asked Simon, an experienced palliative care nurse, to see Doris on the morning of her discharge. Doris unenthusiastically agreed.

### Interpretive Analysis

The 45-minute conversation began when Doris responded to Simon's enquiry how she felt with an account of her illness:

Yeh but erhm (4) all of a sudden I had very sore fingers just before Christmas not this Christmas gone the Christmas previous right (.) and eventually I went to the doctor cos the fingers felt as though you'd dipped them in hot water when it was to::o hot that was all it felt like (coughing)

The care about chronology may indicate an attempt to impose some semblance of rational order on the inchoate experience of illness. Another striking feature of Doris's account is that she presents her cancer illness against a background lifelong good health:

Because I don't really know what pain is you know in a way of speaking I mean I've had five children only one born in hospital during the war the others were all born at home I've never had no problems (.) I didn't want to run up and down hospitals there's no need if you're not if you're not ill is there (.) so you see all this has been rather a surprise but I'd never I've never been ill so it's very difficult

Her resistance to invalid status supports the concept of the ‘sick role’ (Parsons, 1991) and the duty to be healthy, and conforms to a heroic stereotype of ‘fighting’ cancer. Indeed a self-presentational feature of Doris's talk making her story easier to tell is the reference to her individualism and the character trait Doris seems proudest of: stoic self-sufficiency. Doris only goes to the doctor with her painful fingers ‘after everybody said to me go’. When Simon asks what she felt when told she had lung cancer, Doris at first replies: ‘Nothing’, and then invokes the conventional simile: ‘Honestly, I am as tough as old boots’. A few lines later she describes the onset of severe pain at home, again with attention to time:

Not until the well I've been in here just over a week (0.4) about Tuesday I got a (.) I sent my son upstairs to my daughter said for God's sake go and get Valerie I'll have to have a doctor or something (.) he said mother you must be ill (laughs) I said I don't know what it is but I shall be crying in a minute

Her narrative reflects her stoical self-image:

Yes she (Valerie) said to me you're crying mum (laughs) I've never seen you cry (.) I said I can't help it I said it really hu::rts so she got through to the ambulance people

Doris, a ‘tough’ person, cannot bear loss of control caused by the cancer. Another strand of Doris's talk, however, appears to undercut her self-portrayal as a self-reliant, ‘happy-go-lucky’ person who ‘don't mind nothing’ by consistently maintaining a light, bantering tone. She describes how her children speak of her as being a ‘laugh’, repeatedly calls herself ‘a fool’, and significantly refers to her own account of her illness as ‘a pathetic tale’. Admitting that her story is a sad one leads her to speak about her domestic circumstances and how she may manage when she goes home. As the conversation draws to a close the nurse asks if there is ‘anything else you wanted to ask me or anything else you think I should know’. Doris replies at first: ‘No, I don't think so’, then introduces the idea of her death by a euphemistic metaphor:

Well I suppose you will eventually you know when the works come (.) when he said to me without looking into the future I said well you've got a nerve at 75 what do you think there is (laughter and coughing) (0.6)

and possibly attempts to gain mastery over it by talking flippantly of suicide:

Yeh come day go day God sends another day and if he don't send it god help ya (laughter) you know there's nothing you can do about it is there nothing at all I mean we all have to pop-off at some time but I would rather it hadn't been this way there you are it's one of those things isn't it I told them they can all save their money Christmas instead of buying me lots of little presents they can buy me a gun and I'll pop myself off (laughs)

When the nurse asks: ‘What would make you feel like that if the pain got very bad or something or just general sort of thinking about being unwell’, Doris responds:

I don't know (in a low tone) it's just my word (.) just my joke really

Christmas makes a particularly poignant, pathetic backdrop to ‘popping off’. In the image of children saving their Christmas money to buy her a gun she is not simply feeling sorry for herself. Her ability to ‘joke’ is her defence, the idea of approaching death being so frightening that Doris would like to dominate it. When the beginning of the conversation is recalled: ‘All of a sudden I had very sore fingers just before Christmas not this Christmas gone the Christmas previous’, it seems Doris has symbolically woven Christmas into her responses, defensive or self-pitying, to her illness and its possible outcomes. (Christmas is thus a potential entry point for the nurse into the patient's thought processes.)

Up to the very end of the conversation she has been evasive and joking, and has not spoken overtly of death. Perhaps we see here the impact of Simon's skilled approach which has brought her away from concealing metaphoric language and euphemism to talk openly of dying. The nurse reassures Doris that: ‘Whatever happens to you we can keep you there (at home) I'm almost certain of that you know even if things went really wildly wrong.’ At this point Doris abruptly confronts her death, adding: ‘And I came to die’. Understandably, Simon cannot deal with this directly and replies: ‘And you came to have a lot of problems’. Perhaps because of his evasion, Doris significantly retreats to indirect modes of communication, referring pointedly to the weight of her bed-clothes: ‘Like I said I can't stand the weight of this (points to bedclothes) the wei::ght’, recalling her earlier description of avoiding discomfort:

And I find it erhm (.) last night is the only night they brought me up here which I don't remember much about that I've never been under them sheets (whispered) God they are heavy (.) too heavy for me so I propped the pillow up like that so I'm not going to lay down and cough all night and disturb anybody else cos I don't sleep (coughs) and I slipped this over me feet this is ever so warm it keeps your feet ever so warm this wool and I just lay on the top

Her words now acquire added meaning; the weight of the bedclothes may be a metaphor for the dread of dying and being buried.

Doris expresses the unbearableness of her situation covertly through oblique self-reference and idiosyncratic responses (the story of her illness is a ‘pathetic tale’, her cancer a ‘surprise’ and she is ‘tough as old boots’). Her self-pity, conveyed in the image of her children buying her a gun at Christmas, may also lie behind her seemingly incongruous response when Simon asks how she tolerated her radiotherapy treatment: ‘No, I just went up there on me own and came home on me own’. Instead of answering Simon's question about the effects of the treatment, Doris tells of travelling to and from the hospital unaided. Although such self-reliance is not unusual, Doris's gratuitous mention of it, use of the word ‘just’, and repetition of ‘on me own’ suggests not only her need to remain in control of her advanced lung cancer, but an appeal for compassion and admiration.

Another more aggressive side of Doris's feelings is revealed when, after describing her unemployed son's financial difficulties, she abruptly asks: ‘There's something burning here this morning can you smell it (0.6) what are you bu::rning up there.’ Simon tells her it is ‘toast, simple as that’ and Doris replies: ‘Scrape it off and give it to the patients.’

It is as if unconscious resentment surfaced in Doris at her status as a patient and her strange surroundings. Her antagonism is translated into a harsh image of friction (‘scrape it off’) and the resentful ‘give it to the patients’ (i.e. ‘it's good enough for them’). In the context of the whole conversation, the short sharp reply belies Doris's earlier assertion that she felt ‘nothing’ when told she had lung cancer, being ‘as tough as old boots’. ‘Give it to the patients’ denies her own status and projects projection of helplessness onto the other ‘patients’.

Retrospectively, we can now recognize an earlier, more concealed expression of such resistance to patienthood. When Simon wisely accepts her stonewalling assertion that she felt ‘nothing at all’ with ‘So you rather took that information in your stride did you’, Doris replies with both punctual and structural significance: ‘Yes (.) and they gave me urhm radium and I forget when that was’., suggesting she recognizes a rare, precious and valuable element, as a fitting reward for her toughness when facing her diagnosis. Moreover, in the light of her scrupulous attention to chronology elsewhere, ‘forgetfulness’ regarding something that was done to her as a patient seems disdainful, as if to say ‘I attach no importance to it’. The sense depends on symbolic pattern throughout the conversation, one part of which reinforces another in a continuing series of associations that defers its full meaning: the painfulness for the patient of exposing her fear and anger at a perceived sentence of death.

## Patient Two

### Background

These excerpts are from a conversation between Julia, a palliative care support nurse, and Mary, a 59 year-old woman with metastatic breast cancer. Julia has remained in touch with Mary for two years since metastatic spread of her cancer was discovered three months ago. Mary has been undergoing a course of chemotherapy but has become increasingly breathless, a new and frightening experience for her that induces fear of impending death. Mary has sought reassurance from the nurse that something can be done to relieve her breathlessness. Unknown to both Mary and Julia at the time of this 40-minute conversation the cancer has spread to Mary's rib cage and shoulder bones, which collapsed in on her lungs, causing her breathing difficulties.

### Interpretive Analysis

In this excerpt Mary vividly describes her physical experience of the metastatic spread of her cancer, searching for meaning in the face of her worsening symptoms:

I can't can't unbend my shoulders either that's another thing I .why's that(2) my shoulders are all hunched up and I can't I can't get them straight I have to do like a little old (.) hunch back it doesn't straighten out that doesn't help either (.) it's uncomfortable for me (1) radiotherapy wouldn't help me really any more would it … no well on my sort of try to do something to my back you see I go like this (tries to pull her shoulders back) I can't straighten out can I (4) I can't get straight (2.5) oh God I don't know what to do

Mary here achieves a qualitative shift in expression, from describing not being able to straighten her shoulders to metaphorically conveying her all-consuming anxiety at not being able to ‘get straight’, i.e. make sense of her situation, a reading borne out by Mary's desire expressed in the next lines to lead a simple life:

I just want to lead my life (1.8) so simple I don't want I don't want to ask for very much you see (2.2) just want to lead a simple life go to I mean I've got it's a very mundane little job but I li::ke it and (3) and a nice little flat I mean it needs a lot of money spent on it but I like it and I've got what I want and (.) and nothing's too (.) and now all this happens (8) it's .my mouth's so sore I can't eat properly (4)I mean I suppose some of these ulcers are on my throat as well probably (5)which doesn't help

Mary may be attempting to reconcile wants for a ‘simple life’ with what is happening to her: a punishment uncalled for by her modest desires (those who ask too much invite punishment). She may also unconsciously wish to rid herself of her overwhelming feelings of anxiety and discomfort by projecting them onto the nurse. When Julia sympathizes: ‘I know this is really hard for you Mary’, Mary goes on to say: ‘I don't know what to do (1) or say or think (14) sorry it's uncomfortable for you here [leaning on the window ledge] but it's the only way I can’. Mary's solicitude deserves a closer look. It leads Julia to protest: ‘Don't worry I'm fine.’ Julia's sympathy may finally seem too aggressive to Mary since it affirms what Mary does not want to accept. She therefore reacts and temporarily achieves a neat reversal of roles. This externalizing of unacceptable feelings is an important communication which the nurse, with sufficient training and support, could put to use.

Another poignant diversion occurs earlier when Mary mentions her cat and adds: ‘He's 12’ – as cats go, a centenarian. It is a diversion from her own predicament to that of an elderly ‘orphaned’ cat. Mary perhaps envies his longevity while feeling sad that her cat which she loves may be ‘orphaned’. Given the painful nature of the emotion–talk these nuanced switches of topic are not surprising. They also provide a key to Mary's psychic defences which lie not in aggressive rejection and self-presentation of ‘toughness’, but in humility by which Mary tries to maintain moral ascendancy. The meek should not have to leave the world in pain and suffering.

Mary's return to speaking of her discomfort from her mouth and throat ulceration exposes the limitations of Julia's interpretive skills. By complaining that her mouth is so sore she ‘can't eat properly’, Mary may be trying to escape her overwhelming anxiety by evoking primitive feelings of being held and fed (Klein, 1930), her illness provoking regression to infantile dependent behaviour as a defence against the pain of her experience. It appears very difficult for the nurse to decipher Mary's anxious communication; she seems to play along with Mary's evasive action, acknowledging the symptoms to which Mary refers and reassuring her that they can be alleviated, saying in effect: ‘That we can cure’. Long conversational pauses suggest that Mary may want the nurse to think for her but Julia's last response to Mary's questions about the possibility of further treatment:

Yeh I hope so (1) try something (treatment) (8) it might be that they could try some radiotherapy (4) I don't know whether they'd be able to do that tomorrow .it's probably unlikely but erm that might help

indicates that, on the contrary, Julia herself does not ‘know what to do or say or think’ when faced by Mary's sudden deterioration and infantile projections.

## Patient Three

### Background

This final conversation reveals other aspects of the idiosyncratic nature of a patient's emotional expression. The 40-minute talk takes place between Lorraine, a relatively inexperienced ward nurse, and Helen, a woman of 70 who nursed her husband after a stroke until his death several years ago and now fears a recurrence of her breast cancer, diagnosed five years previously. All necessary investigations to account for her jaundice have been carried out but Helen has not yet been told the results, which she will learn at the next Tuesday medical ward round.

### Interpretive Analysis

Helen has been voicing her worry about the test results, using euphemistic metaphors for cancer, e.g. ‘hiccups in my breast’ and ‘this thing raised its ugly head’, and also describing her fear that ‘they may find something that is detrimental to me’. Having observed women on the ward having chemotherapy Helen is very frightened by what she has seen, particularly the sickness some of the women experienced. She thinks it is inevitable that she will have to have such an ‘unnatural treatment’. Her anxieties about chemotherapy and its side effects may be related to the further loss of control over her life and body, which the treatment and further hospitalization would inevitably mean. Helen raises the issue of control in the following extract, eight minutes into the conversation:

I would like to go home so that I can sort of erhm (.) have my own things and my own surroundings and take care of my own destiny (laughs) well in as much how things are going to go and do I have to adapt to anything after all the treatment (.) you know your lifestyle can be changed can't it it can be overnight I mean if someone has a car accident and they lose an arm or a leg your destiny changes and you have to adapt to all that

When Lorraine asks her to elaborate on what she means by her ‘destiny’, Helen distances herself from the subject by using the second or third person to avoid contemplating her own future. Lorraine perhaps recognizes this evasiveness: she repeats her question and asks Helen what would be her ‘worst destiny’. Helen does give a direct response to the question which is directly followed by a rejection of this destiny:

The worst .mm I suppose if I was confined just to my own flat (.) disablement I suppose (.) I think (3) I er it just wouldn't be me at all (2) I'd sooner look after someone else if you see what I mean yes well erhm yes in his (husband's) case he was looking back he was an excellent patient because I've seen patients at the Brook (hospital) and some of them were cruel to their wives because they were ill I don't want to be like that with my family I hope that I if I have to say goodbye to them I hope I'm still sensible enough to make them understand you know (3.2) that's all (.) I just don't want them hurt (.) but there you go (.) there we go (1) but I don't think anyone can help you on that because (.) in the sense of erhm they can be patient with you and er kind to you and so on but it's inside really isn't it that sort of thing one of those things (2) so how do you think I should approach Tuesday you tell me

Helen's response can be understood in several ways. One reading is that the thought of her death arouses very negative feelings and destructive ideas; she associates helplessness with cruelty and hurt. These ideas are framed by euphemisms for death: ‘if I have to say goodbye’, ‘but there you go there we go’ and ‘one of those things’. Before changing the subject, Helen localises her feelings ‘inside’ her inner world, beyond the help of others. Helen seeks some respite from considering the gravity of her situation by asking the nurse how she should approach Tuesday but does not wait for an answer:

Well would you be scared if you were having this that sort of thing (.) I mean would you be anxious or I know you see sometimes it's worse when you have more knowledge doesn't it and you have knowledge and experience on this (1) and er no I just wondered how you would say to a person to change that idea you know but I should be all right I'll be as good as gold (laughter) with all those strong men around you and wanting to get you better how could you do otherwise but er it's just the build-up it's the build-up if you were a sportsman and you or a rugby team or whatever it's the build-up to erhm er (nurse sneezes) oops you've still got your cold haven't you oh no

While hope can be kept up throughout the illness and dying process (e.g. hope for a successful operation, hope for a peaceful death at home) without necessarily denying the illness, some forms of denial seem involved in clinging to hope and a determination not to let the illness ‘win’ (Emanuel *et al*., 1990). It is in this sense that Helen may be appealing to the nurse to tell her how to face Tuesday's ward round. Here the nurse did not take the easy option of reassuring the patient but neither did she facilitate further discussion as she did when she asked Helen to identify her worst destiny. Instead Lorraine sneezes, providing an anti-climax to the ‘build-up’. The confused syntax of Helen's conversation just before the sneeze reflects Helen's anxiety and fear. Helen seems to be comparing her suspenseful situation to that of a rugby team before a match – perhaps a metaphor for the aggressive ‘game’ being played against the cancer by her medical team and the destructive feelings that she may have been in touch with during their conversation.

In this case it is not so much a matter of disclosing feelings through the personal symbolism of metaphor as of metaphor-making itself as an activity to deal with unbearable reality and provide the patient a measure of relief. The metaphors may be original and humorous (e.g. ‘the hiccup’ or finding something ‘detrimental’ earlier in the conversation); more often they are trite euphemisms – ‘say goodbye’. ‘There you go’ is an interesting example of a dead metaphor – ‘there you are’ – suddenly changing as Helen unconsciously adapts it to her situation, shielding herself behind ‘you’, then the first person plural ‘we’. This happens again with ‘that sort of thing’, given a twist to the more light-hearted ‘one of those things’. ‘Tuesday’ itself becomes a metaphor for ‘hearing the truth’. The search for metaphorical expressions intensifies –hence the confused syntax – after the nurse gives Helen free rein to express her anxiety. The old cliché ‘good as gold’ enables her to laugh by its inappropriateness, a line she pursues with the ironic, trite feminine image of ‘strong men around you wanting you to get better’; then she seeks comfort in the metaphor of a sportsman's or whole rugby team's ‘build-up’, to a match, the match in her case being ‘Tuesday’), until the nurse accidentally or from some psychological mechanism of her own punctures the metaphorical balloon by a down-to-earth sneeze. Helen's metaphor-making suddenly stops: below she becomes simply aware, not of a rugby team's situation, but of her own:

It's just the build-up to the day isn't it I think that's all I've got really (coughs) I tell myself that you know tell myself that but it's nice when other people talk to you and er allow you to get a little bit off your chest sort of thing I appreciate it Lorraine very much yes of course I do darling yep I do I do I do yes yeh (1) about my worries I haven't got any worries really I'm very lucky I'm very very lucky

Bereft of metaphor Helen turns to Lorraine (whom she may think that ‘getting a little off her chest’ has caused to sneeze) as a refuge where metaphors are unnecessary. But of course as soon as she returns to the subject of her illness the metaphoric activity starts up again:

but as regards the illness I think I think I've told you all about it really because there's not a lot to report at this stage of the game I mean if (.) if er I mean the doctors are very honest with you and I think you appreciate that (.) course you do

It may be that metaphor here is not so much a vehicle for hidden feelings but a protection against them. This patient finds distraction and relief in playing with and parodying metaphor.

## Discussion

### Summary Findings

Our study revealed emotion disclosure of intensity and relentlessness. Emotional expression pervades patients' dialogue with nurses during day-to-day care. Some patients express emotions very directly to their nurses; others use less direct forms of communication involving complex and difficult language, frequently in symbolic and metaphorical form. Nurses in the study used an impressive range of responsive strategies to enable patients to talk about their feelings, even when this involved the risk of sustaining the talk and escalating its intensity. However, they did not consistently facilitate emotional expression and at times evaded the patients' attempts to disclose. Nurses often did not identify and respond to indirect expressions of feeling contained in the figurative aspects of patients' talk.

### Figurative Language Use

Our results concur with those of previous studies that confirm routine use of metaphor by patients to express their distress and the demands of the cancer illness (Gwyn, 2002; Radley, 1993) making use of their bodies as a rich source of metaphor (Gordon, 1990; Kirmayer, 1992). Our analysis also suggests that in some situations such expression, while apparently avoiding emotion, is symbolized in safer, more bearable metaphoric form (Winnicott, 1965). This unconscious mode of truth-telling allows the patient to approach their feelings but to keep at a distance so as not to be overwhelmed by them. This finding complements other studies showing that some cancer patients often refrain from giving, or are unable to give, direct expression to their emotions, especially negative ones like anger, depression and hostility (Greer & Watson, 1987). Some patients suffer from alexithymia and are unable to find appropriate words to describe their feelings at all. Methodologically sound evidence for the role of alexithymia in cancer patients is needed and studies are required to investigate its consequences, if any, on cancer incidence and progression (De Vries *et al*., 2012). Our study highlights the risk for patients who may rely on figurative expression – that their psychological distress may not be appropriately responded to or treated. Metaphoric expression of feelings introduced ambiguity and indirectness into patients' talk possibly leading to misinterpretation or meaning being missed by the nurse (Reyna, 1996) who may be ‘excused’ from pursuing the expression since the metaphor is ambiguous.

A number of writers have recognized the metaphoric potential of cancer illness (Penson *et al*., 2004; Sontag, 1979). Although cancer patients' figurative language use has not been hitherto a primary focus of study, Stacey, drawing on her own experience as a patient, concluded that the acute anxiety generated by cancer creates a climate and need for metaphoric thinking: ‘(The) fear which the naming of cancer engenders fuels the desire to seek linguistic reassurance: perhaps cancer does not have to be confronted if we do not speak its name’ (Stacey, 1997, p. 63). Metaphors, she maintains, may come to the rescue: by transforming one thing into another they can enable patients to acknowledge their situation without confronting and reflecting on its unbearableness (see our interpretation of Doris's conversation with Simon). Metaphors allow cancer patients to approach the truth obliquely (e.g. to come close to the contemplation of death, as with Doris) and then to distance themselves in a way perhaps less threatening to them, and to the nurse (Parkes, 1978). Figurative language use is thus seen as a necessary part of the patient's defensive system. Sontag (1979) took a different line, namely that certain dominant metaphor, particularly of war, dangerously constrained and controlled how emotions were expressed in cancer care. In our study, metaphors of war, e.g. ‘they attacked it with chemotherapy’, and ‘my natural defences are low’ were prevalent, but they did not completely exclude forbidden topics such as the cancer illness and possible death, as Sontag originally suggested. Although overt expression of these subjects may be avoided in a cancer ward, they remain in everyone's mind and are only partially disguised by the metaphors.

Our study consolidates and adds to previous knowledge of unconscious defence mechanisms used by cancer patients, e.g. splitting, projection and introjection (Guex, 1994). To our knowledge it is the first to focus on the defensive function of figurative language in patient–nurse conversations.

### Patient–Nurse Interaction

Studies of patient–nurse interaction are rare (Jarrett & Payne, 2000). Our findings revealed that emotional disclosure was neither solely determined by the patient nor wholly initiated and controlled by the nurse. Instead, the joint contributions of the nurse and patient in the give-and-take of the conversation had a vital bearing on the articulation of emotion, modifying the assumption that nurses control and influence the progress of their conversations with patients' with ‘blocking’ verbal behaviours to avoid emotionally charged subjects and further patient disclosure (Maguire *et al*., 1996; Wilkinson, 1991). Our study shows that if nurses ward off certain topics, so do patients. They also create contexts for sustaining their disclosure, sometimes despite discouragement by the nurse. These findings shift the emphasis firmly away from the nurse as sole agent managing disclosure and towards mutual nurse–patient interaction in those processes.

The case excerpts transcribed above typify conversations across the whole data set of 60 recorded conversations and reveal the extent of the challenge nurses face in their day-to-day talk with patients. The nurses in the study were exposed to almost continuous expression of powerful feelings often in complex language and metaphor. The guarded nature of nurses' talk found in this study is not unique, but an important stable phenomenon in the field, tempered here by nurses' use of facilitative communication skills. The reason invariably given for it is that nurses lack the communication skills, confidence and ongoing support to elicit patient concerns and feelings and help them examine their emotional responses (Sheldon *et al*., 2009). Our results provide additional reasons, given below.

### Impact on the Nurse

Exposure to patients' symbolic and metaphoric expression exposes nurses directly to their own unconscious fears and anxieties. Nurses' inability or failure to acknowledge emotion expression, apparent throughout the literature, indicates it might be too powerful an experience, the more so because of its frequency. Menzies Lyth (1988) suggests that nurses may be deeply defended against the stresses and anxieties of their work. Institutional and home care settings and nurses' physical care role may facilitate t this defence, imposing time and other constraints that permit only superficial care; the nurse moreover is working in situations where imminent death is often a very real possibility, leaving no opportunity to revisit distressing topics raised by the patient. These features of the nurses' work contrast markedly with the psychotherapists' boundaried therapeutic space and planned session work, providing opportunities for reflection, further exploration of difficult feelings and a chance to verify meaning.

Some nurses in this study were palliative care nurses (see case study 2), seeing patients at home as well as in hospital. Their experience was not framed by the relatively ‘protective’ environment of hospital wards and departments. On a home visit the nurse, often alone, enters the patient's world and life and cannot fail to be aware of the patient's change from a former self, thus magnifying the recognition of loss. The nurse is especially vulnerable to the powerful transference feelings and projections from patients and their families which may be felt very personally (i.e. the nurse becomes a ‘good’ or ‘bad’ person for the patient and family or is made to assume different roles). Dominant nursing models of nursing as a rational, problem-solving, practical activity contribute to the nurses' difficulty (Roper *et al*., 2000). Working within mainly objectifying, bio-medical techno-rational cultures, the nurses may not surprisingly have difficulty in understanding patients' metaphors – an abstract, reflective, interpretive, literary activity for which they are not trained. Our study foregrounds the working conditions of nurses and their relentless exposure to patients' feelings: the nurse must attend to serious problems in the patient's external physical world which may relate to their own psychic world. Nurses may have defences against understanding because of their own unresolved conflicts and fears, particularly those to do with loss and death. In a classic paper Main (1957) considers that nurses' choice of work with cancer has deep personal reasons and that it has abiding unconscious determinants such as the need to heal sick parts of themselves. A range of feelings, therefore, may invade the nurse as she works, including anxiety, guilt, depression and compulsive reparative wishes (Barnes *et al*., 1998; Goldie, 2005).

## Implications

The richness of the patients' articulation of feeling in personal imagery, metaphor and symbol in the study raises important questions for nurse training and support. An illness conceived in metaphoric and symbolic terms might be responded to in kind. Does such interpretive work lie within the nurses remit and, if so, what do cancer nurses and their educators need to do? What is appropriate to teach, and to whom? Cognitive-based communication skills training is likely to fall short in preparing cancer nurses to deal with the complexities revealed by this study, especially if it does not include a follow-up element to facilitate and sustain the use of newly acquired skills (Heaven, Clegg & Maguire, 2006). Education and training should include support for understanding the emotional experience of both the nurse and the patient and their complex interaction. Any form of training is likely to be limited in effect without skilled supervision (Morton-Cooper & Palmer, 2000).

The level of projected distress and emotion disclosed to nurses by patients in this study raises concerns about the nature of support and supervision required by nurses to maintain full receptivity to the emotional needs and suffering of patients. Cancer patients are extremely vulnerable and therefore often form a close relationship with their nurse caregiver. The part that patterns of attachment play in forging this relationship has been revealed in the pioneering work of Bowlby (1982) and Parkes, Stevenson-Hinde & Marris (1991). Early relationships and the memory of their affective quality, especially between the child and its primary caregiver, influence present relationships including that between patient and nurse (Fonagy *et al*., 2007). Insecure attachment orientations in the past can lead to potentially distressful interactional and relational patterns, particularly in the context of cancer illness. Knowledge of the attachment style may provide important clues to the nurse as to how a patient may use her or him psychologically (Braun *et al*., 2011; Rodin *et al*., 2007). How the attachment style and the organizing principles of patients may affect their experience of health care providers and their ability to accept and obtain emotional support is a potential practice tool for the nurse (Stolorow & Atwood, 1992, pp. 24–5).

Wright, McMahon and Pearson (1991) highlighted the physical and psychological exhaustion involved in nurses' therapeutic work. They questioned lay and professional expectations that nurses work to develop close therapeutic relationships with patients without a personal and managerial commitment to support and train the nurse and provide containment for the transference and projections they receive. Nurses need career-long supervision and support to understand and bear disclosure by patients, including their figurative communications. They also need help to understand how the experience of patients meshes with their own lives and experience. Supervision is now a requirement for clinical nurse specialists in cancer and palliative care (National Institute for Clinical Excellence [NICE], 2004). A culture of clinical supervision does not yet exist in nursing and implementation of the NICE policy is patchy, although it has been regarded as essential in the health care setting (Nissim *et al*., 2012). Where it has been implemented, the frequency and quality of supervision and its uptake are highly variable. Some nurses are proactive and seek supervision; some establish Balint groups (Savage, 2004), while others may be reluctant to take up supervision, fearing that it reflects an inability to cope with the emotional demands of the job (Kelly, Long & McKenna, 2001). The crucial question whether supervision can help nurses overcome and bear the difficulties of their work for patient benefit requires further research.

## Research Implications

Although there is substantial evidence that psychological therapies and interventions to facilitate emotional expression and discussion of feelings affect psychological outcomes in cancer, there is no robust evidence that they affect survival rates (Sheinfeld Gorin *et al*., 2012). A number of comprehensive reviews of the research evidence have been conducted including meta-analyses in which results from multiple small scale studies are combined and statistically analysed to identify patterns and relationships have concluded that no robust randomized controlled trial has been designed with survival as an end-point, where psychotherapy has not been confounded with medical care and treatment. Trials have not yielded a positive result (Petticrew, Bell & Hunter, 2002). Chida *et al*. (2008) caution that their meta-analysis shows the protective effects of positive psychological well-being on cancer survival and mortality need to be interpreted with caution due to different therapeutic interventions used, small sample sizes, and the variety of measures used to assess outcome. Coyne *et al*. (2007) examined whether emotional well-being predicted survival in a large sample of patients with head and neck cancer participating in multicentre clinical trials. Their results add to the evidence that emotional functioning is not an independent predictor of survival in cancer patients. Our study shows that articulating feelings is an imperative for cancer patients interacting with the nurses who care for them and that additional prospective research studies are needed with stringent recruitment, explanatory variable ascertainment, outcome variable ascertainment and fully controlled co-variates. Linking positive psychological outcomes to biological measures will be key in future research.

## Conclusion

Our research highlights the multiple challenges involved in emotion talk in cancer and provides important insights into cancer patients' individual use of figurative language and metaphor in emotional expression during day-to-day care. It provides evidence of the unconscious processes involved in nurses' work. Psychoanalytically orientated supervision and support for nurses is necessary to enhance their ability to engage in challenging dialogue with cancer patients.
